# Effects of initial boost with TGF-beta 1 and grade of intervertebral disc degeneration on 3D culture of human annulus fibrosus cells

**DOI:** 10.1186/s13018-014-0073-8

**Published:** 2014-08-14

**Authors:** Aldemar Andres Hegewald, Jessie Cluzel, Jan Philipp Krüger, Michaela Endres, Christian Kaps, Claudius Thomé

**Affiliations:** 1 Department of Neurosurgery, University Medical Center Mannheim, Heidelberg University, Theodor-Kutzer-Ufer 1-3, Mannheim 68167, Germany; 2 TransTissue Technologies GmbH, Charitéplatz 1, Berlin 10117, Germany; 3 Tissue Engineering Laboratory, Department of Rheumatology, Charité University Medicine Berlin, Charitéplatz 1, Berlin 10117, Germany; 4 Department of Neurosurgery, Innsbruck Medical University, Anichstr. 35, Innsbruck 6020, Austria

**Keywords:** Tissue engineering, Regenerative medicine, Spine, Intervertebral disc, Annulus fibrosus, Biomaterial, Growth factors

## Abstract

**Background:**

Three-dimensional (3D) culture in porous biomaterials as well as stimulation with growth factors are known to be supportive for intervertebral disc cell differentiation and tissue formation. Unless sophisticated releasing systems are used, however, effective concentrations of growth factors are maintained only for a very limited amount of time in *in vivo* applications. Therefore, we investigated, if an initial boost with transforming growth factor-beta 1 (TGF-beta 1) is capable to induce a lasting effect of superior cartilaginous differentiation in slightly and severely degenerated human annulus fibrosus (AF) cells.

**Methods:**

Human AF tissue was harvested during surgical treatment of six adult patients with lumbar spinal diseases. Grading of disc degeneration was performed with magnet resonance imaging. AF cells were isolated and expanded in monolayer culture and rearranged three-dimensionally in a porous biomaterial consisting of stepwise absorbable poly-glycolic acid and poly-(lactic-co-glycolic) acid and a supportive fine net of non-absorbable polyvinylidene fluoride. An initial boost of TGF-beta 1 or TGF-beta 1 and hyaluronan was applied and compared with controls. Matrix formation was assessed at days 7 and 21 by (1) histological staining of the typical extracellular matrix molecules proteoglycan and type I and type II collagens and by (2) real-time gene expression analysis of aggrecan, decorin, biglycan, type I, II, III, and X collagens as well as of catabolic matrix metalloproteinases MMP-2 and MMP-13.

**Results:**

An initial boost with TGF-beta 1 or TGF-beta 1 and hyaluronan did not enhance the expression of characteristic AF matrix molecules in our 3D culture system. AF cells showed high viability in the progressively degrading biomaterial. Stratification by grade of intervertebral disc degeneration showed that AF cells from both, slightly degenerated, or severely degenerated tissue are capable of significant up-regulations of characteristic matrix molecules in 3D culture. AF cells from severely degenerated tissue, however, displayed significantly lower up-regulations in some matrix molecules such as aggrecan.

**Conclusions:**

We failed to show a supportive effect of an initial boost with TGF-beta 1 in our 3D culture system. This underlines the need for further investigations on growth factor releasing systems.

## Background

In the interventional and surgical treatment of degenerative intervertebral disc diseases, there is a need for annulus fibrosus (AF) repair techniques because they address the unsolved problem of re-herniation through the untreated annulus defect after intervertebral disc herniation [1] and they facilitate the development of nucleus pulposus augmentation techniques by providing adequate nucleus containment [2].

Major components of the AF are collagens (60%–70% of dry weight), mostly type I collagen, and proteoglycans (15%–20% of dry weight) [3]. These matrix molecules were shown to have considerably low turnover rates [4],[5]. Age and degeneration appear to have distinguishable effects on the AF [6]–[8]. The overall proteoglycan concentration, including the small proteoglycans decorin and biglycan, seem to decrease with age; whereas with degeneration, increased proteoglycan concentrations for moderate and decreased proteoglycan concentrations for severely degenerated AF are reported. In addition, AF degeneration is associated with increased type I and III collagen synthesis, an increased expression of inflammatory factors and matrix-degrading molecules, as well as with a deregulation of anabolic factors [3],[9]. These changes result in a catabolic metabolic state with increasing fibrous transformation, impaired biomechanical function, and the incidence of AF defects. For biological annular repair techniques, the choice of a biocompatible and biomechanically suitable material is crucial [10]. But the additional enhancement of tissue generation with bioactive factors might be another important component [11], regardless of whether a cell-free [12] or a cell-based approach [13],[14] is applied.

In a previous work, we reported on the suitability of a polymer-based absorbable textile scaffold to facilitate the expression of characteristic matrix molecules of AF cells in three-dimensional (3D) culture [15]. Moreover, we showed an anabolic effect of human serum, FGF-2, and transforming growth factor-beta (TGF-beta) 3 on AF cells with significantly enhanced expression of characteristic matrix molecules [11].

In this work, we tested a textile porous biomaterial consisting of stepwise absorbable poly-glycolic acid (PGA) and poly-(lactic-co-glycolic) acid (PLGA) and a supportive fine net of non-absorbable polyvinylidene fluoride (PVDF). The concept of using an absorbable polymer-based biomaterial, supported by a fine PVDF net for primary stability, revealed promising biomechanical and biocompatibility characteristics in a cell-free approach for AF defects in an ovine model [16]. Here, the utility of the PGA-PLGA-PVDF scaffold for a cell-based approach with human AF cells from slightly and severely degenerated lumbar intervertebral discs was investigated. Since TGF-beta has shown promising results as an anabolic factor for AF cells [11], we examined the potential of an initial boost with TGF-beta with and without hyaluronan to enhance characteristic matrix formation.

## Materials and methods

### Human annulus fibrosus cell preparation and cultivation

Human annulus fibrosus tissue was harvested during surgical treatment of spondylolistesis (*n* = 4) or scoliosis (*n* = 2). Grading of disc degeneration was performed using preoperative magnet resonance imaging (MRI) as described previously [17]. Three out of six donor tissues were graded as ‘slightly degenerated’ (*n* = 3 females age 33–43; mean age 40 years, spinal segments: L4/L5, L5/S1, L5/S1); three out of six donor tissues were graded as ‘severely degenerated’ (*n* = 3 females, age 38–72; mean age 61 years, spinal segments: L3/L4, L4/L5, L5/S1). The ethics committee of University Medical Center Mannheim approved the study, and informed patient consent was obtained.

The tissue was minced and digested for 2 h at 37°C in Dulbecco's modified Eagle's (DME) medium (Biochrom, Berlin, Germany) containing 10% human serum (German Red Cross, Berlin, Germany), 2 ng/ml fibroblast growth factor (PeproTec, Hamburg, Germany), 1.5 U/ml collagenase P (Roche, Basel, Switzerland), 500 U/ml collagenase CLS type II (Biochrom), 50 U/ml hyaluronidase (Sigma, St. Louis, MO, USA), 100 U/ml penicillin (Biochrom), 100 mg/ml streptomycin (Biochrom) in a spinner flask under gently stirring. The resulting cell suspension was poured through a nylon cell strainer (250 μm; BD Falcon, Franklin Lakes, NJ, USA) and centrifuged at 585 × *g*. The cell pellet was washed twice with DME medium and phosphate-buffered saline (PBS). The annulus fibrosus-derived cells (AF cells) were counted using trypan blue (Sigma) dye exclusion and seeded into cell culture flasks with an initial density of 8,000 cells/cm^2^. AF cells were expanded in DME medium supplemented with 10% human serum and antibiotics as described above. Medium was changed every 2 to 3 days. After reaching 90% confluence, AF cells were detached with trypsin/EDTA solution (Biochrom) and subcultured with a seeding density of 8,000 cells/cm^2^.

### 3D culture of annulus fibrosus-derived cells

Textile porous scaffolds (3T GmbH, Aachen, Germany) made of layers of absorbable PGA, PLGA, and PVDF were sterilized by gamma irradiation (approx. 30 kGy). PGA-PLGA-PVDF scaffolds (5 mm × 10 mm × 1 mm) were loaded with either 50 μl sterile water (empty scaffold serving as control), 2.5 ng transforming growth factor-®1 (TGF-beta 1) in 50 μl sterile water (TGF-beta 1 scaffold), or 2.5 ng TGF-beta 1 in 50 μl hyaluronan (10 mg/ml, OSTENIL, TRB Chemedica AG, Munich, Germany) designated TGF-beta 1-HA scaffold. Scaffolds were freeze-dried for 16 h using a lyophilisator (Leybold-Heraeus, Stuttgart, Germany) and stored in a desiccator at room temperature.

Expanded (passage 3) slightly and severely degenerated AF cells (pool of *n* = 3 cell preparations derived from individual donors, each) were detached. PGA-PLGA-PVDF scaffolds were loaded with 1.0 × 10^6^ AF cells mixed with fibrinogen (Tissucol, Baxter, Westlake Village, CA, USA). Fibrinogen was polymerized by addition of thrombin (1:10 *v*/*v* in PBS) for 20 min at 37°C. TGF-beta 1 scaffolds loaded with slightly (*n* = 4) and degenerated (*n* = 4) AF cells, TGF-beta 1-HA scaffolds loaded with slightly (*n* = 4) and degenerated (*n* = 4) AF cells, and empty scaffolds loaded with slightly (*n* = 4) and degenerated (*n* = 4) AF cells were cultured in 6-well plates for up to 21 days in DME medium supplemented with 5% human serum, 100 U/ml penicillin, and 100 mg/ml streptomycin. Medium was exchanged every 2–3 days.

### Real-time gene expression analysis

As described previously [18], total RNA were isolated from slightly and severely degenerated AF cells expanded in monolayer cultures (passage 3) and from AF cells cultured in 3D PGA-PLGA-PVDF scaffolds loaded with TGF-beta 1 or TGF-beta 1-HA or in empty scaffolds. Equal amounts of total RNA (1 μg) were transcribed into single-stranded cDNA using the iScript Kit according to the manufacturer's recommendations (BIO RAD, Munich, Germany). The relative expression of the housekeeping gene glyceraldehyde-3-phosphate dehydrogenase (GAPDH) was used to normalize the cDNA samples. One microliter of each normalized cDNA sample was analyzed using the SYBR Green PCR Core Kit (Applied Biosystems, Darmstadt, Germany) and a real-time PCR Cycler (iCycler; BIO RAD). Expression levels of genes coding for extracellular matrix molecules and matrix modifying enzymes (Table 1) were analyzed, and the fold change (FC) was calculated according to the ΔΔCT method [19], in comparison to AF cells cultured in empty PGA-PLGA-PVDF scaffolds or to expanded monolayer AF cells. A FC of ≤ −2 or ≥2 was considered as differential expression.

**Table 1:** Oligonucleotide sequences

**Gene name**	**Accession number**	**Oligonucleotides (5′ → 3′), (up/down)**	**Base pairs**
Aggrecan	NM_001135	GGC TGC TGT CCC CGT AGA AGA/GGG AGG CCA AGT AGG AAG GAT	163
Biglycan	NM_001711	GCT GCC CCT GCT CTC CCA CCA CA/GAA ATG CAT GAG GAG GAG GAA CAG AAC	217
Collagen type I	NM_000088	CGA TGG CTG CAC GAG TCA CAC/CAG GTT GGG ATG GAG GGA GTT TAC	180
Collagen type II	NM_001844	CCG GGC AGA GGG CAA TAG CAG GTT/CAA TGA TGG GGA GGC GTG AG	128
Collagen type III	NM_000090	GGT TTT GCC CCG TAT TAT GGA/AGT TTC TAG CGG GGT TTT TAC GAG	142
Collagen type X	NM_000493	GAA CTC CCA GCA CGC AGA ATC C/GTG TTG GGT AGT GGG CCT TTT ATG	145
Decorin	NM_001920	ACT TCT GCC CAC CTG GAC ACA ACA C/AAT GGC AGA GCG CAC GTA GAC ACA	128
Glyceraldehyde-3-phospate dehydrogenase	NM_002046	GGC GAT GCT GGC GCT GAG TAC/TGG TCC ACA CCC ATG ACG A	149
Matrix metalloproteinase 2	NM_004530	TCC CTG CCC CTC CCT TCA AC/CCT TTC CAG CAG ACA CCA TCA CC	196
Matrix metalloproteinase 13	NM_002427	CAA AAA CGC CAG ACA AAT GTG ACC/GAT GCA GGC GCC AGA AGA ATC T	105

### Histology and immunehistochemistry

For histochemical and immunehistochemical analysis of proteoglycans and types I and II collagen, AF cells in PGA-PLGA-PVDF scaffolds were embedded in optimum cutting temperature (OCT) compound, frozen and cryo-sectioned (6 μm). Proteoglycans were stained at pH 2.5 with Alcian blue 8GX (Roth, Germany), followed by counterstaining with nuclear fast red (Sigma). Collagens and proteoglycans were visualized by staining with 0.7% Safranin O (Sigma) and counterstaining with 0.2% Fast Green (Sigma). For detection of collagens, slides were incubated with rabbit anti-human type I collagen or anti-human type II collagen antibodies (both Acris, Heford, Germany) for 40 min. Colorimetrical detection was done with 3-amino-9-ethylcarbazole (EnVision™, Dako, Glostrup, Denmark) and counterstaining with hematoxylin (Merck, Darmstadt, Germany). To evaluate AF cell viability and distribution within the scaffolds, propidium iodide/fluorescein diacetate (PI/FDA) staining (Sigma) was performed as described [20]. Scaffolds loaded with AF cells were analyzed using a fluorescent microscope (Olympus AX70, Hamburg, Germany).

### Statistics

Gene expression data were analyzed using SigmaStat 3.5 (Systat Software, Erkrath, Germany). The parametric *t* test and the non-parametric Mann-Whitney rank sum test were applied. Differences were considered significant at *p* < 0.05.

## Results

**Figure 1:**
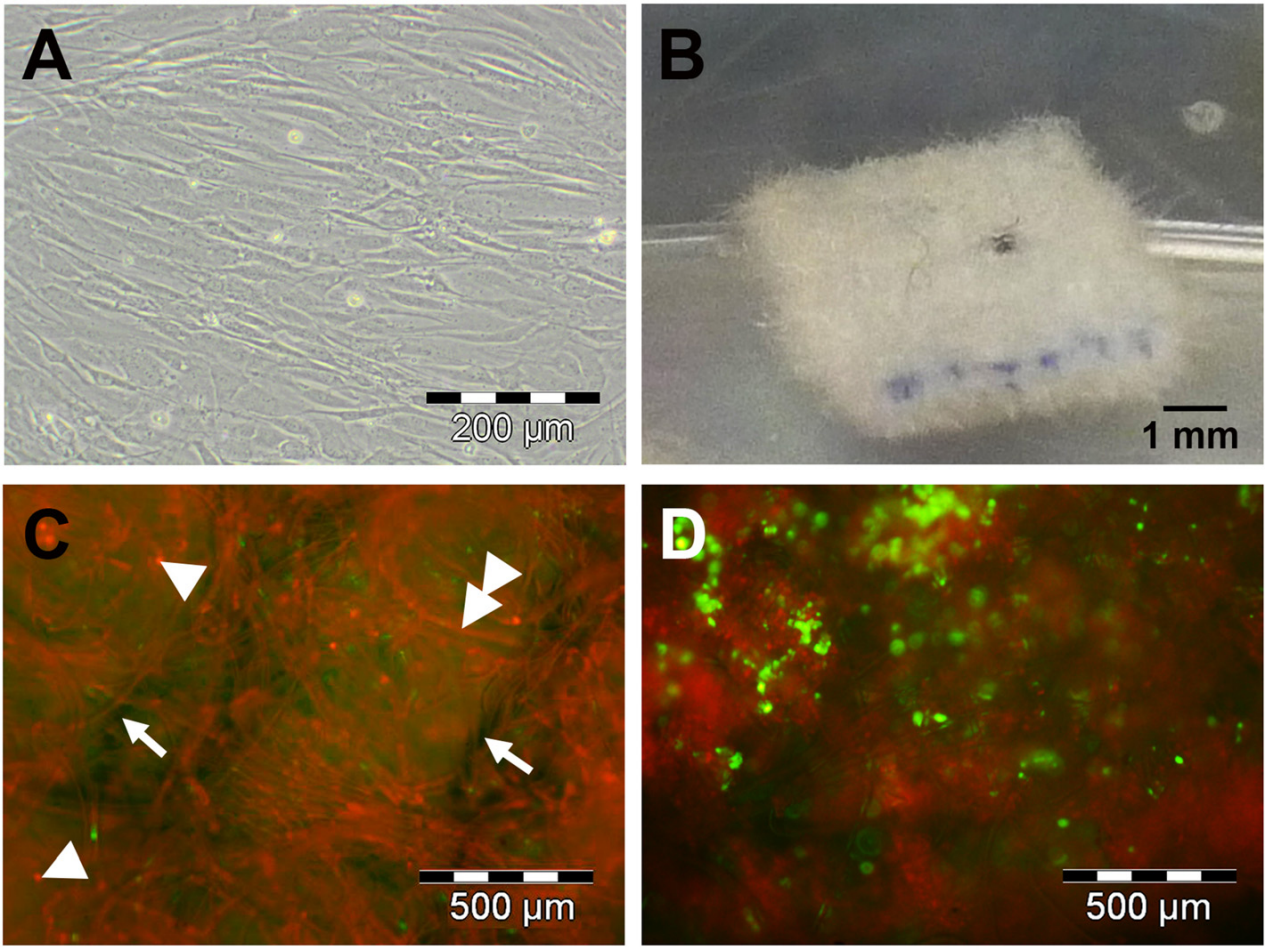
**Tissue culture of expanded AF cells in 3D scaffolds.** Expanded AF cells showed a stretched, fibroblastic morphology when cultured in monolayers up to passage 3 **(A)**. AF cells were embedded in PGA-PLGA-PVDF scaffolds **(B)** and maintained viability (green staining) after 7 **(C)** and 21 days **(D)** of tissue culture. At day 7, some dead cells (**C**, white arrowheads) were evident. Absorbable scaffold fibers (**C**, white double arrowhead) showed signs of degradation as early as day 7 and took the red propidium-iodide dye (red staining). The non-absorbable PVDF fibers took no dye and showed a dark blue color (**C**, white arrows).

Human AF cells derived from slightly and severely degenerated annulus fibrosus tissue were grown in the presence of human serum, using standard cell and tissue culture techniques (Figure 1). During monolayer cell culture and expansion, cells began to stretch and showed the typical fibroblast-like morphology of dedifferentiated chondrocytic cells (Figure 1A). To initiate re-differentiation, expanded cells (passage 3) were seeded in textile 3D polymer scaffolds made of PGA-PLGA and PVDF (Figure 1B) and cultured for 3 weeks. Life/dead staining (FDA/PI) at day 7 (Figure 1C) and day 21 (Figure 1D) of tissue cultures revealed mostly viable cells indicated by green staining and only few red stained cells indicating cell death (Figure 1C, white arrowhead). Increasing red staining (PI) by polymer fibers indicated progressive degradation of absorbable polymer fibers (Figure 1C, white double arrowhead). The non-absorbable PVDF fibers did not take up the dye and showed a dark bluish color (Figure 1C, white arrow).

### Effect of initial boost with TGF-beta 1 or TGF-beta 1-hyaluronan

**Figure 2:**
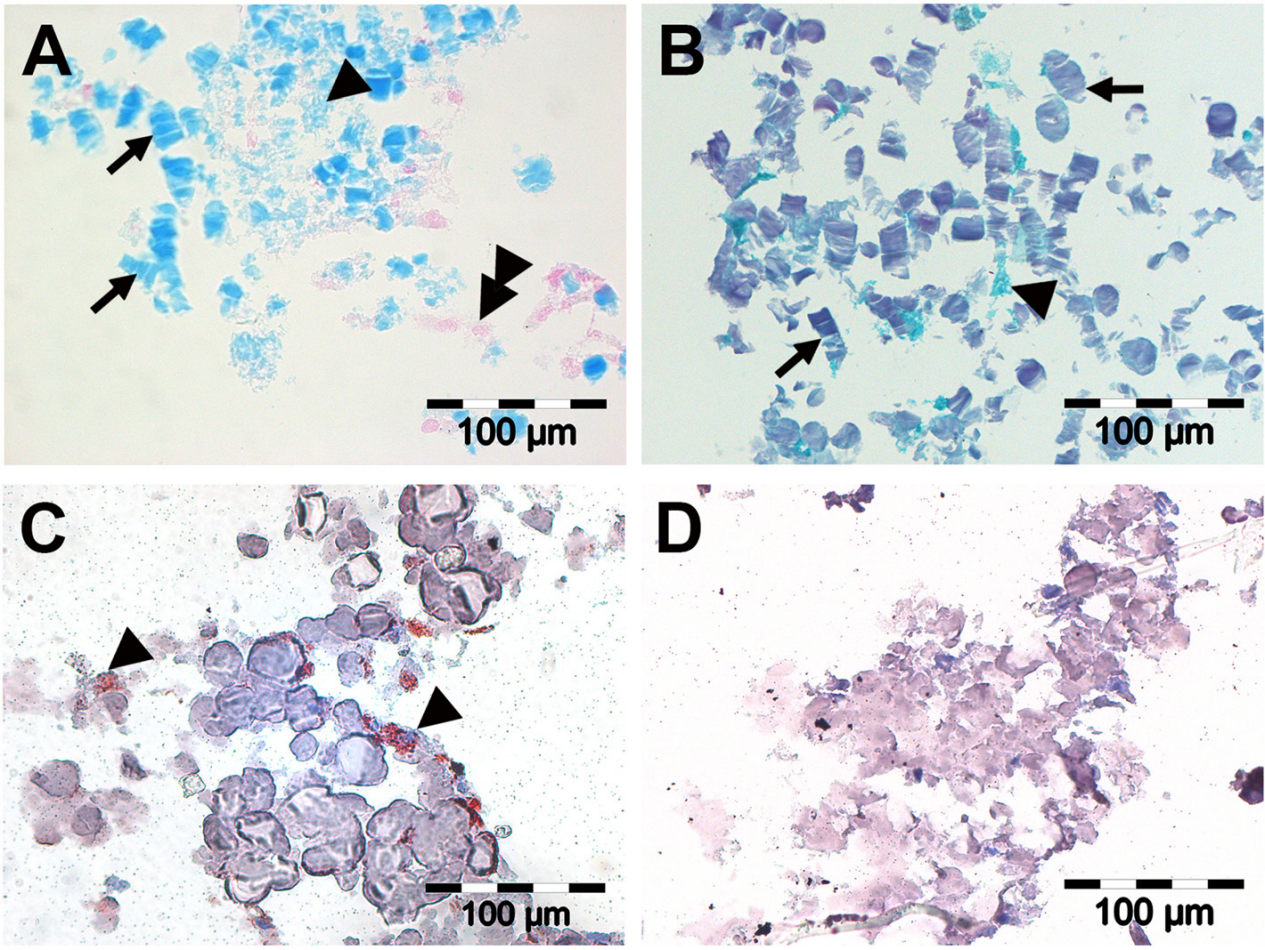
**Histological and immunehistochemical staining of AF cells cultured in 3D scaffolds.** At day 21, alcian blue staining **(A)** was weak and showed marginal deposition of proteoglycans (**A**, black arrowhead), but there was no pronounced staining of the matrix surrounding AF cells (**A**, double arrowhead). Safranin O staining showed no proteoglycans but a weak staining of a collagenous matrix (**B**, black arrowhead). Absorbable scaffold fibers took the staining dye and appeared fragmented as a sign of degradation (**A**, **B**, black arrows). AF cells stained positively for type I collagens (**C**, black arrowheads), while staining for type II collagens were negative **(D)**.

Expanded AF cells were seeded in empty 3D scaffolds and scaffolds loaded with TGF-beta 1 (50 ng/ml) or TGF-beta 1 (50 ng/ml)-hyaluronan and cultured for 3 weeks without further addition of TGF-beta 1. Histological analysis of 3D scaffolds seeded with AF cells showed that AF cells marginally formed a cartilaginous matrix after 21 days of 3D culture (Figure 2), independent of an initial boost with TGF-beta 1 or TGF-beta 1-hyaluronan (data not shown). Alcian blue staining of proteoglycans showed some deposition of proteoglycan (Figure 2A, black arrowhead), but there was no pronounced staining of extracellular matrix around AF cells (Figure 2A, black double arrowhead). Safranin O showed weak staining of a collagenous matrix but no proteoglycans (Figure 2B, black arrowhead). The scaffold fibers were fragmented and showed typical signs of degradation (Figure 2A,B, black arrow). Immune staining showed collagen type I (Figure 2C, black arrowhead), but no deposition of collagen type II (Figure 2D). Isotype controls (IgG) were negative and confirmed specificity of the immune staining (data not shown).

**Figure 3:**
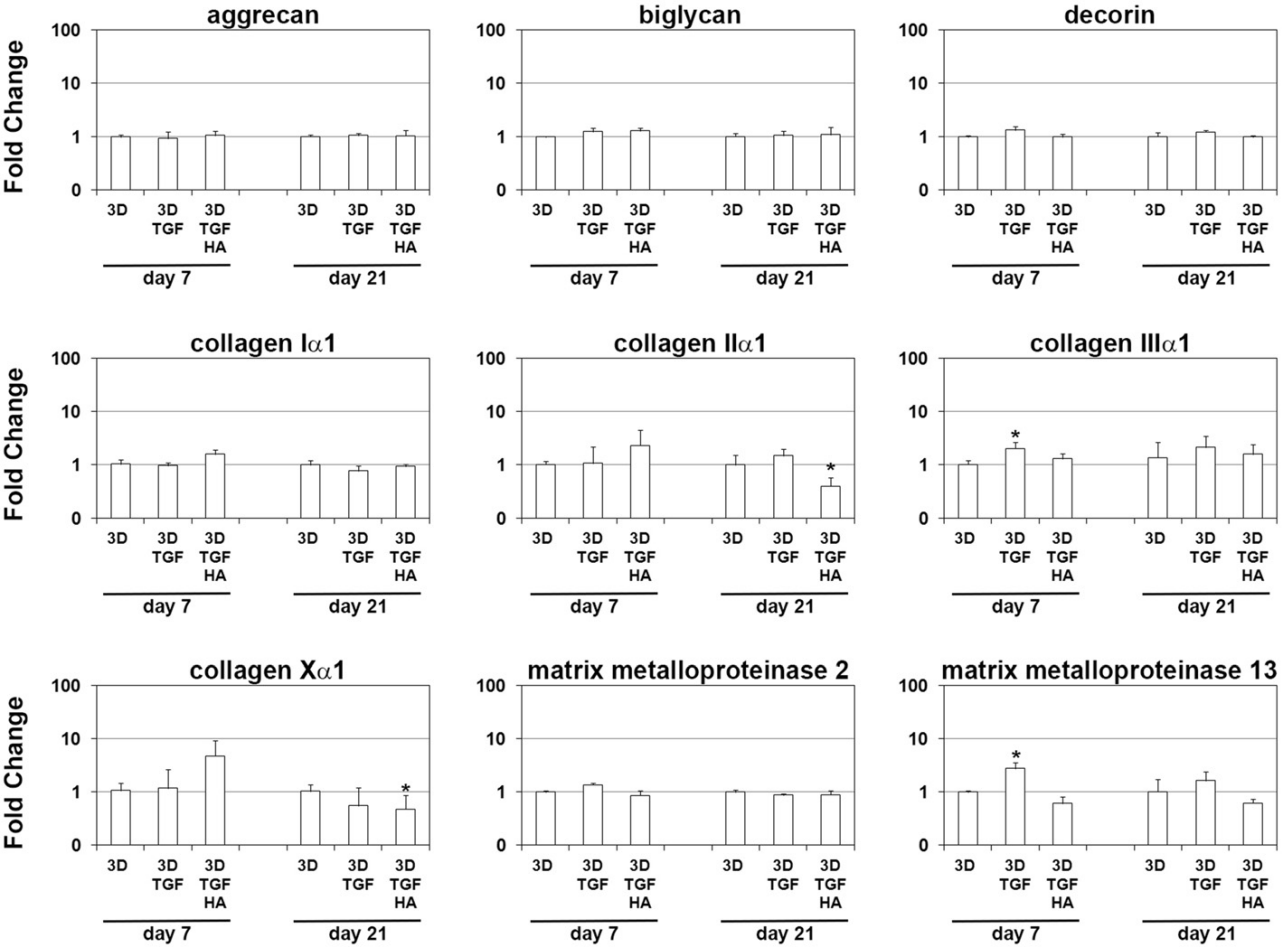
**Gene expression analysis of AF cells cultured in 3D scaffolds.** Gene expression analysis of AF cells in empty PGA-PLGA-PVDF scaffolds (3D), scaffolds loaded with TGF-beta 1 (3D TGF), or TGF-beta 1-hyaluronan (3D TGF HA) was performed after 7 and 21 days of tissue culture. Fold changes of genes coding extracellular matrix molecules and matrix modifying enzymes are given compared to their expression level found in AF cells cultured in empty scaffolds at day 7 or day 21. Bars show the mean of each triplicate and standard deviation. Asterisks (*) indicate significant differential expression (*p* < 0.05).

Gene expression analysis of characteristic AF matrix molecules and matrix modifying enzymes was performed at day 7 and day 21 of 3D culture (Figure 3). The initial boost with TGF-beta 1 on AF cells seeded in the 3D polymer scaffolds did not influence the expression profile of aggrecan, biglycan, decorin, collagen types I, II and X, as well as matrix metalloproteinase (MMP) 2, compared to non-stimulated 3D cultures. At day 7, but not at day 21, initial boost stimulation with TGF-beta 1 significantly (*p* < 0.05) induced the expression of collagen type III (fold change, FC = 2.0) and MMP13 (FC = 2.8), compared to non-stimulated controls. After prolonged culture for 21 days, initial boost stimulation with TGF-beta 1-hyaluronan significantly (*p* < 0.05) repressed the expression of collagen type II (FC = −2.5) and collagen type X (FC = −2.1), compared to non-stimulated controls.

### Effect of grade of intervertebral disc degeneration on AF cell re-differentiation in 3D culture

**Figure 4:**
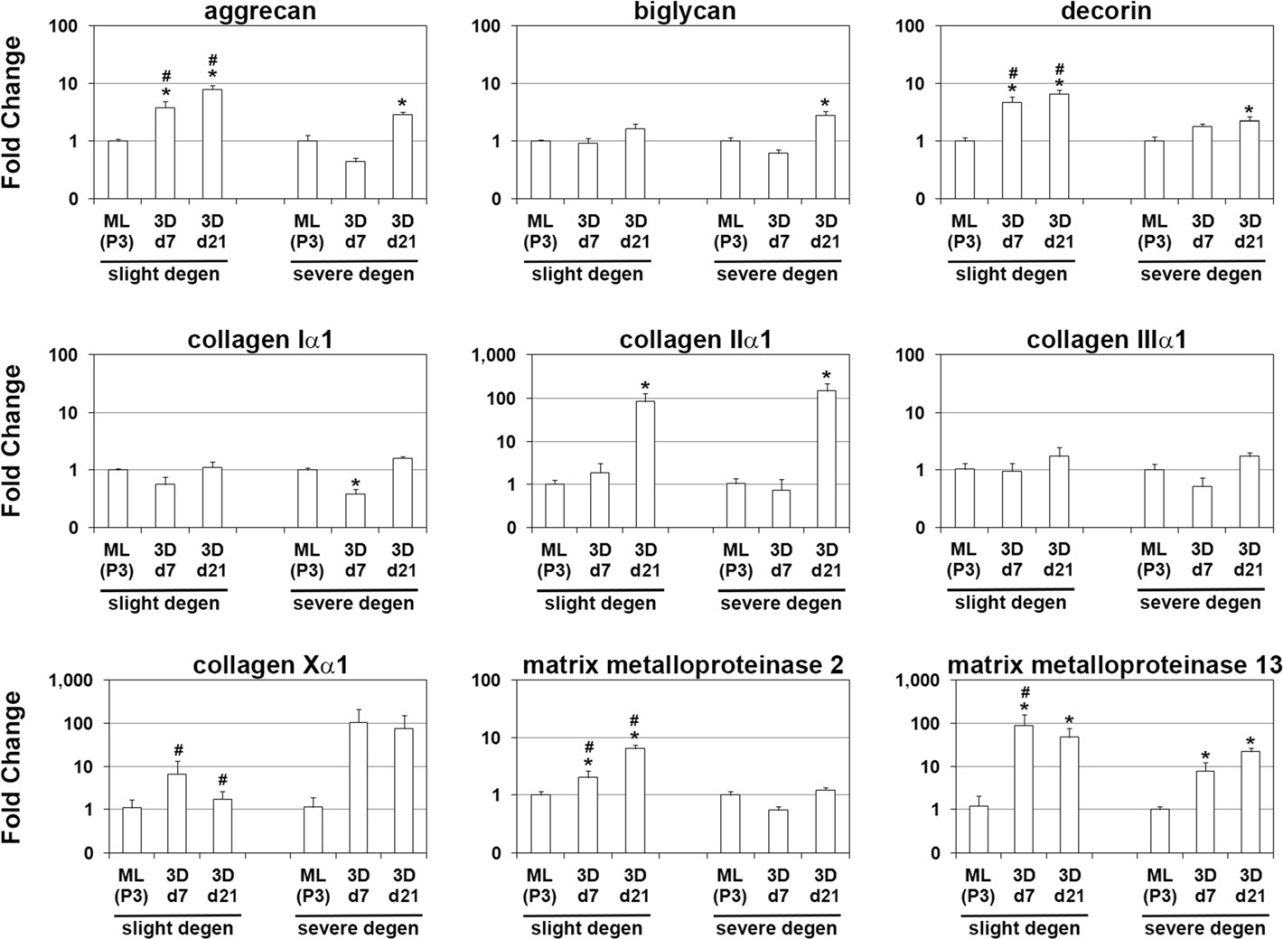
**Gene expression analysis of slightly and severely degenerated AF cells cultured in 3D scaffolds.** Gene expression data were stratified by degeneration status of AF cells and are given for slightly and severely degenerated AF cells cultured in monolayers and in PGA-PLGA-PVDF scaffolds (3D) for up to 21 days. Fold changes of genes coding extracellular matrix molecules and matrix modifying enzymes are given compared to their expression level found in monolayer expanded AF cells. Bars show the mean and standard deviation. Asterisks (*) indicate significant differential expression (*p* < 0.05) compared to monolayer cultures. Number sign (#) indicate significant differential expression (*p* < 0.05) compared to severely degenerated AF cells.

To analyze the effect of annulus tissue degeneration on the re-differentiation capacity of expanded AF cells in 3D polymer scaffolds, gene expression profiles were stratified by degeneration status. The expression levels of extracellular matrix marker genes found in AF cells cultured in polymer scaffolds were compared to levels found in monolayer cultures (Figure 4). 3D assembly of expanded AF cells derived from slightly degenerated annulus fibrosus tissue in polymer scaffolds resulted in significant induction (*p* < 0.05) of the expression levels of aggrecan (up to FC = 7.8 at day 21), decorin (up to FC = 6.5 at day 21), collagen type II (FC = 83.4 at day 21), MMP2 (up to FC = 6.4 at day 21), and MMP13 (FC = 89.4 at day 7 and FC = 47.2 at day 21), compared to the expression levels found in expanded AF cells in monolayer from slightly degenerated tissue. The induction of the expression levels of aggrecan, decorin, MMP2, and MMP13 in slightly degenerated AF cells in 3D polymer scaffolds was significantly higher than the induction found in severely degenerated AF cells cultured in 3D polymer scaffolds, while the collagen type X induction was significantly lower.

Severely degenerated AF cells cultured in 3D polymer scaffolds showed significant induction of the expression levels of aggrecan (FC = 2.9 at day 21), biglycan (FC = 2.8 at day 21), decorin (FC = 2.2 at day 21), collagen type II (FC = 146.5 at day 21), and MMP13 (up to FC = 21.7 at day 21), compared to severely degenerated AF cells cultured in monolayer. At day 7, but not at day 21, the expression of collagen type I was significantly reduced in severely degenerated AF cells in 3D polymer scaffolds (FC = −2.6), compared to monolayer controls.

## Discussion

The PGA-PLGA-PVDF scaffold under investigation in this study showed good biocompatibility in terms of AF cell viability in 3D culture after 7 and 21 days. Progressive degradation of the absorbable scaffold components, however, was already observed as early as day 7. In conjunction with the observation of low AF protein deposition, deduced from the weak staining results of characteristic matrix molecules, it is unclear, whether this construct would be biomechanically stable enough to facilitate AF repair in a clinical application. Several biomaterials have been suggested for AF repair that, in general, showed good biocompatibility with AF cells *in vitro*[10]. With regard to absorbable biomaterials, however, optimal absorption time with associated biomaterial stability has not been investigated systematically. At gene expression levels, we found a general differentiation of the expanded dedifferentiated AF cells toward a cartilaginous expression profile. But the more pronounced expression of aggrecan and type II collagen is actually more suggestive for nucleus pulposus-like tissue than for AF tissue. This is probably the result of missing biomechanical forces *in vitro*. Several studies indicate that especially intermittent loading patterns on AF cell cultures can modulate more characteristic AF expression profiles [21],[22].

In patients with degenerative disc diseases, slightly and severely degenerated intervertebral discs can be observed. Here, we investigated if the grade of degeneration influences the gene expression profile in our 3D culture system. Characteristic matrix molecules type I, II and III collagen as well as aggrecan were assessed by gene expression analysis. The large proteoglycan aggrecan that provides a water-retaining chemical structure for the intervertebral disc was expressed in significantly higher amounts in AF cells from slightly degenerated discs compared to AF cells from severely degenerated discs. Collagen type I, II and III, however, did not display any differences in this regard. In addition, the small proteoglycans biglycan and decorin were assessed. These bind to collagens, growth factors, and other matrix components and thereby appear to regulate the assembly of matrix and to influence repair processes after injury [7],[23]. In this study, Decorin, but not Biglycan, displayed significant higher gene expression levels in AF cells from slightly degenerated discs. A potentially undesirable response is type X collagen expression, which is a marker for chondrocyte hypertrophy that might lead to tissue calcification or even ossification [24]. Type X collagen expression of AF cells from slightly degenerated tissue was found to be significantly lower than in AF cells from severely degenerated discs. The matrix metalloproteinases MMP-2 and MMP-13 were previously found to be involved in degenerative processes of the intervertebral disc [25],[26]. Here, they were analyzed to assess catabolic processes. We observed significantly higher expression levels of MMP-2 and MMP-13 in AF cells from slightly degenerated discs compared to AF cells from severely degenerated disc. It is not clear, however, whether MMP induction in our 3D culture system is an undesirable effect or part of a natural balancing response to the induced anabolic processes that, in general, appeared to be more pronounced in AF cells from slightly degenerative discs.

TGF-beta has shown promising results as an anabolic factor for AF cells *in vitro*[11],[27]. In *in vivo* applications, however, growth factors maintain effective concentrations only for a very limited amount of time. We hypothesized that an initial boost with TGF-beta could induce a lasting effect of superior cartilaginous differentiation that could even be further enhanced with hyaluronan. Hyaluronan is a major component of the intervertebral disc matrix that was shown to support chondrogenic differentiation of mesenchymal stem cells in previous reports [28],[29]. In this 3D culture system, however, an initial boost of TGF-beta 1 with or without hyaluronan did not enhance anabolic gene expression profiles and failed to induce a superior cartilaginous matrix formation. A weakness of this study is that we tested only one growth factor in one concentration. Higher concentrations for the initial boost might yield better results but also carry the risk of undesired neurological complications *in vivo*[30]. Other possible strategies to provide longer lasting effects of bioactive factors *in vivo* could be growth factor releasing systems [13],[31], gene therapeutic approaches, or simply a longer pretreatment with bioactive factors *in vitro*.

## Conclusions

We failed to show a supportive effect of an initial boost with TGF-beta 1 for AF cell differentiation and tissue formation in our 3D culture system. This underlines the need for further investigations on growth factor releasing systems. Expanded AF cells from severely degenerated intervertebral disc tissues revealed the capacity to re-differentiate toward a cartilaginous gene expression profile in 3D culture and displayed only minor inferiority compared to AF cells from slightly degenerated tissue in this regard. This might qualify AF cells from severely degenerated tissue to be also considered for cell-based therapeutical approaches.

## Abbreviations

3D: three-dimensional

AF: annulus fibrosus

FGF: fibroblast growth factor

MRI: magnet resonance imaging

PGA: poly-glycolic acid

PI/FDA: propidium iodide/fluorescein diacetate

PLGA: poly-(lactic-co-glycolic) acid

PVDF: polyvinylidene fluoride

TGF-beta: transforming growth factor-beta

## Competing interests

J. Cluzel, J. P. Krüger, M. Endres, and C. Kaps are employees of TransTissue Technologies GmbH, which develops products in the field of regenerative medicine. A. A. Hegewald and C. Thomé declare that they have no competing interests.

## Authors’ contributions

AAH contributed to the conception and design of the study and of the PGA-PLGA-PVDF scaffold. He drafted part of the manuscript. JC performed the cell culture experiments and histological analysis. JPK took part in the histological and gene expression analyses. ME took part in the histological and gene expression analyses and supervised cell culture experiments. CK contributed to the conception and design of the study and drafted part of the manuscript. CT critically revised the clinical aspects of the study. All authors read and approved the final manuscript.
